# Sodium piperidine-1-carbodithio­ate dihydrate

**DOI:** 10.1107/S1600536811022604

**Published:** 2011-06-18

**Authors:** Ana C. Mafud, Maria Teresa P. Gambardella

**Affiliations:** a Instituto de Química de São Carlos, Universidade de São Paulo, Av. Trabalhador Sãocarlense 400, Caixa Postal 780, 13560-970, São Carlos, SP, Brazil

## Abstract

The asymmetric unit of the title compound, Na^+^·C_6_H_10_NS_2_^−^·2H_2_O, is composed of a sodium cation, a piperidine­dithio­carbamate anion which exhibits positional disorder, and two lattice water mol­ecules. The atoms of the piperidine ring are divided over two sites with occupancy factors of 0.554 (6) and 0.446 (6). In the crystal, the sodium cation (coordination number of 6) and the piperidine­dithio­carbamate anion are linked, forming an infinite two-dimensional network extending parallel to (001). O—H⋯S hydrogen bonds, involving the lattice water mol­ecules, also aid in stabilizing the crystal sructure.

## Related literature

For the crystal structures of similar compounds, see: Oskarsson *et al.* (1979 ▶); Albertsson *et al.* (1980 ▶); Ymén (1982 ▶); Mafud & Gambardella (2011 ▶). For puckering parameters, see: Cremer & Pople (1975 ▶). 
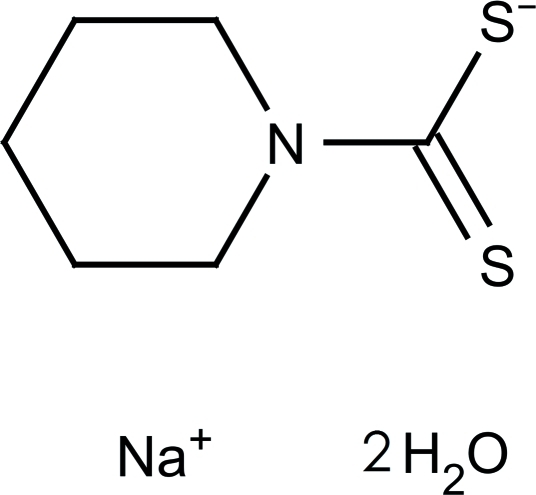


## Experimental

### 

#### Crystal data

Na^+^·C_6_H_10_NS_2_^−^·2H_2_O
*M* *_r_* = 219.29
Monoclinic, 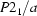

*a* = 12.241 (5) Å
*b* = 5.909 (5) Å
*c* = 14.690 (5) Å
β = 95.519 (5)°
*V* = 1057.6 (11) Å^3^
*Z* = 4
Mo *K*α radiation
μ = 0.51 mm^−1^
*T* = 290 K
0.02 × 0.02 × 0.02 mm

#### Data collection

Nonius KappaCCD diffractometer
7553 measured reflections
1863 independent reflections
1482 reflections with *I* > 2σ(*I*)
*R*_int_ = 0.145

#### Refinement

*R*[*F* ^2^ > 2σ(*F* ^2^)] = 0.054
*wR*(*F* ^2^) = 0.152
*S* = 1.02
1863 reflections
176 parameters
6 restraints
H atoms treated by a mixture of independent and constrained refinement
Δρ_max_ = 0.33 e Å^−3^
Δρ_min_ = −0.36 e Å^−3^

### 

Data collection: *COLLECT* (Nonius, 1998 ▶); cell refinement: *SCALEPACK* (Otwinowski & Minor, 1997 ▶); data reduction: *DENZO* (Otwinowski & Minor, 1997 ▶) and *SCALEPACK*; program(s) used to solve structure: *SIR92* (Altomare *et al.*, 1994 ▶); program(s) used to refine structure: *SHELXL97* (Sheldrick, 2008 ▶); molecular graphics: *ORTEP-3 for Windows* (Farrugia, 1997 ▶) and *Mercury* (Macrae *et al.*, 2006 ▶); software used to prepare material for publication: *WinGX* (Farrugia, 1999 ▶) and *publCIF* (Westrip, 2010 ▶).

## Supplementary Material

Crystal structure: contains datablock(s) I, global. DOI: 10.1107/S1600536811022604/su2283sup1.cif

Structure factors: contains datablock(s) I. DOI: 10.1107/S1600536811022604/su2283Isup2.hkl

Supplementary material file. DOI: 10.1107/S1600536811022604/su2283Isup3.cml

Additional supplementary materials:  crystallographic information; 3D view; checkCIF report

## Figures and Tables

**Table 1:** Hydrogen-bond geometry (Å, °)

*D*—H⋯*A*	*D*—H	H⋯*A*	*D*⋯*A*	*D*—H⋯*A*
O1—H11*O*⋯S2^i^	0.84 (2)	2.39 (2)	3.214 (2)	167 (3)
O1—H12*O*⋯S1^ii^	0.85 (2)	2.49 (2)	3.322 (3)	167 (3)
O2—H21*O*⋯S2^ii^	0.83 (2)	2.48 (2)	3.283 (2)	163 (3)
O2—H22*O*⋯S1^iii^	0.86 (2)	2.46 (2)	3.313 (2)	174 (3)

Symmetry codes: (i) 
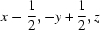
; (ii) 
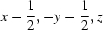
; (iii) 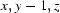.

**Fig. 1.**
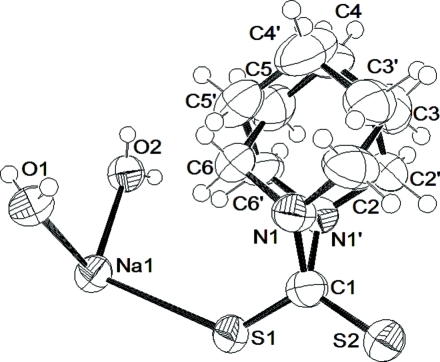
Perspective view of asymmetric unit of the molecular structure of the title compound, with the numbering scheme and displacement ellipsoids drawn at the 50% probability level. The two components (A = C2',C3',C4',C5',N1' and B = C2,C3,C4,C5,N1) of the disordered piperidine ring are shown.

**Fig. 2.**
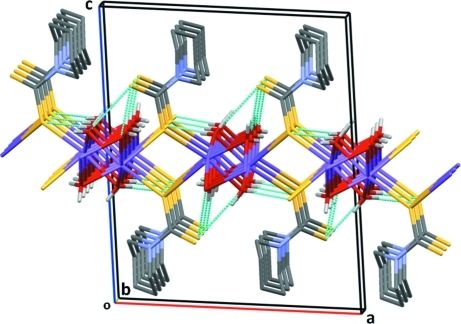
A view along the b-axis of the crystal packing of the title compound. Only the minor (B) component of the disordered piperidine ring is shown. The O-H···S hydrogen bonds are shown as dashed cyan lines and the C-bound H atoms have been omitted for clarity.

## supplementary crystallographic information

### Comment

The title compound is compossed of a piperidinedithiocarbamate anion, a sodium cation, and two lattice water molecules (Fig. 1). The crystal structures of similar compounds, for example sodium 1-pyrrolidinecarbodithioate dihydrate, has been reported on previously (Oskarsson *et al.*, 1979; Albertsson *et al.*, 1980; Ymén, 1982). The crystal structure of ammonium piperidine-1-carbodithioate dihydrate has been descibed by our group recently (Mafud & Gambardella, 2011).

The atoms of the piperidine ring are disordered, occupying two positions (A = C2',C3',C4',C5',N1' and B = C2,C3,C4,C5,N1) with occupancies of 0.554 (6)/0.446 (6). Both of these six-membered rings have a chair conformation, with puckering parameters, Q = 0.552 (13) Å, θ = 180.0 (13) °, φ2 = 128 (25) °, for ring A, and Q = 0.577 (15) Å, θ = 0.0 (15) °, φ2 = 313 (27) °, for ring B (Cremer & Pople, 1975).

The sodium atoms are coordinated to two sulfur atoms [Na1···S1 3.0649 (15) Å and Na1···S1^i^ 2.9644 (15) Å; symmetry code: (i) -*x*+3/2, *y*-1/2, -*z*+1] and four oxygens [Na1···O2 2.360 (3) Å, Na1···O1 2.385 (2) Å, Na1···O1^ii^ 2.416 (3) Å, Na1···O2^iii^ 2.515 (2) Å; symmetry codes: (ii) -*x*+1, -*y*, -*z*+1; (iii) -*x*+1, -*y*-1, -*z*+1], with a bi-pyramidal reversed geometry. This configuration generates close packed layers which remain cohesive in crystal stacking by van der Waals interactions. The distances of these contacts are slightly less than the sum of the van der Waals radii.

In the crystal O-H···S hydrogen bonds, involving the lattice water molecules, aid in stabilizing the crystal structure (Table 1). The crystal packing gives rise to a supramolecular structure, whose infinite two-dimensional network, with base vectors: #1 = [0 1 0], #2 = [1 0 0], grows parallel to (001), as shown in Fig. 2.

### Experimental

The title compound was prepared by slow addition of 0.1 mol of CS_2_ to a cold solution containing 0.2 mol of piperidine and a stoichiometric amount of sodium hydroxide in ethanol/water 1:1 (*v*/*v*). The reaction mixture was placed in the freezer for 12 h and then filtered through a Büchner funnel, washed with cold ether and the product recrystallized in an ethanol water mixture 1:1 (*v*/*v*). Colourless single crystals, suitable for X-ray diffraction analysis, were obtained. On heating they sublimed and decomposed.

### Refinement

The H-atom positions of the water molecules were located in a difference Fourier map, they were refined with distance restraints, O-H = 0.84 (2) Å, with *U*_iso_(H) = 1.5*U*_eq_(parent O-atom). The C-bound H-atoms of the anion were included in calculated positions and treated as riding atoms: C—H = 0.97 Å, with *U*_iso_(H) = 1.2*U*_eq_(parent C-atom).

### Crystal data

**Table:**

Na^+^·C_6_H_10_NS_2_^−^·2H_2_O	*F*(000) = 464
*M**_r_* = 219.29	*D*_x_ = 1.377 Mg m^−^^3^
Monoclinic, *P*2_1_/*a*	Mo *K*α radiation, λ = 0.71073 Å
Hall symbol: -P 2yab	Cell parameters from 24030 reflections
*a* = 12.241 (5) Å	θ = 2.9–26.7°
*b* = 5.909 (5) Å	µ = 0.51 mm^−^^1^
*c* = 14.690 (5) Å	*T* = 290 K
β = 95.519 (5)°	Prism, colourless
*V* = 1057.6 (11) Å^3^	0.02 × 0.02 × 0.02 mm
*Z* = 4	

### Data collection

**Table:**

Nonius KappaCCD diffractometer	1482 reflections with *I* > 2σ(*I*)
Radiation source: Enraf Nonius FR590	*R*_int_ = 0.145
graphite	θ_max_ = 25.1°, θ_min_ = 3.3°
Detector resolution: 9 pixels mm^-1^	*h* = −14→14
CCD rotation images, thick slices scans	*k* = −7→6
7553 measured reflections	*l* = −17→16
1863 independent reflections	

### Refinement

**Table:**

Refinement on *F*^2^	Primary atom site location: structure-invariant direct methods
Least-squares matrix: full	Secondary atom site location: difference Fourier map
*R*[*F*^2^ > 2σ(*F*^2^)] = 0.054	Hydrogen site location: inferred from neighbouring sites
*wR*(*F*^2^) = 0.152	H atoms treated by a mixture of independent and constrained refinement
*S* = 1.02	*w* = 1/[σ^2^(*F*_o_^2^) + (0.0964*P*)^2^] where *P* = (*F*_o_^2^ + 2*F*_c_^2^)/3
1863 reflections	(Δ/σ)_max_ < 0.001
176 parameters	Δρ_max_ = 0.33 e Å^−^^3^
6 restraints	Δρ_min_ = −0.36 e Å^−^^3^

### Special details

**Table:**

Geometry. All e.s.d.'s (except the e.s.d. in the dihedral angle between two l.s. planes) are estimated using the full covariance matrix. The cell e.s.d.'s are taken into account individually in the estimation of e.s.d.'s in distances, angles and torsion angles; correlations between e.s.d.'s in cell parameters are only used when they are defined by crystal symmetry. An approximate (isotropic) treatment of cell e.s.d.'s is used for estimating e.s.d.'s involving l.s. planes.
Refinement. Refinement of *F*^2^ against ALL reflections. The weighted *R*-factor *wR* and goodness of fit *S* are based on *F*^2^, conventional *R*-factors *R* are based on *F*, with *F* set to zero for negative *F*^2^. The threshold expression of *F*^2^ > σ(*F*^2^) is used only for calculating *R*-factors(gt) *etc*. and is not relevant to the choice of reflections for refinement. *R*-factors based on *F*^2^ are statistically about twice as large as those based on *F*, and *R*- factors based on ALL data will be even larger.

### Fractional atomic coordinates and isotropic or equivalent isotropic displacement parameters (Å^2^)

**Table:**

	*x*	*y*	*z*	*U*_iso_*/*U*_eq_	Occ. (<1)
S1	0.74854 (5)	0.03097 (13)	0.39216 (4)	0.0481 (3)	
S2	0.86593 (6)	0.24242 (13)	0.24400 (5)	0.0577 (3)	
Na1	0.57813 (8)	−0.24210 (16)	0.48568 (8)	0.0509 (4)	
O1	0.41274 (14)	−0.0826 (4)	0.41396 (13)	0.0542 (5)	
H11O	0.403 (3)	−0.012 (5)	0.3644 (17)	0.081*	
H12O	0.369 (2)	−0.194 (4)	0.418 (2)	0.081*	
O2	0.55132 (14)	−0.5918 (4)	0.40928 (13)	0.0533 (5)	
H21O	0.509 (2)	−0.604 (6)	0.3618 (16)	0.08*	
H22O	0.604 (2)	−0.687 (5)	0.409 (2)	0.08*	
C1	0.7694 (2)	0.0599 (5)	0.27824 (18)	0.0551 (7)	
N1	0.6797 (6)	0.0084 (12)	0.2149 (4)	0.0541 (16)	0.446 (6)
C2	0.6746 (8)	0.0673 (16)	0.1177 (5)	0.081 (3)	0.446 (6)
H2A	0.73	0.1807	0.1092	0.097*	0.446 (6)
H2B	0.6034	0.1336	0.0991	0.097*	0.446 (6)
C3	0.6917 (19)	−0.128 (5)	0.0585 (18)	0.095 (8)	0.446 (6)
H3A	0.7657	−0.1862	0.0716	0.114*	0.446 (6)
H3B	0.6818	−0.0832	−0.0053	0.114*	0.446 (6)
C4	0.6069 (10)	−0.314 (3)	0.0773 (10)	0.079 (4)	0.446 (6)
H4A	0.5339	−0.2616	0.0555	0.095*	0.446 (6)
H4B	0.6218	−0.449	0.043	0.095*	0.446 (6)
C5	0.6089 (7)	−0.3753 (16)	0.1787 (6)	0.076 (2)	0.446 (6)
H5A	0.5505	−0.4818	0.1877	0.091*	0.446 (6)
H5B	0.6784	−0.4451	0.1998	0.091*	0.446 (6)
C6	0.5931 (7)	−0.1592 (18)	0.2320 (6)	0.067 (2)	0.446 (6)
H6A	0.5213	−0.0958	0.2135	0.08*	0.446 (6)
H6B	0.5969	−0.1932	0.2969	0.08*	0.446 (6)
N1'	0.7338 (4)	−0.1085 (10)	0.2187 (3)	0.0516 (13)	0.554 (6)
C2'	0.7582 (5)	−0.1213 (13)	0.1240 (4)	0.0677 (19)	0.554 (6)
H2'1	0.7906	−0.267	0.1125	0.081*	0.554 (6)
H2'2	0.8103	−0.0042	0.1119	0.081*	0.554 (6)
C3'	0.6545 (14)	−0.091 (5)	0.0626 (11)	0.095 (6)	0.554 (6)
H3'1	0.6717	−0.1086	−0.0002	0.115*	0.554 (6)
H3'2	0.6291	0.0629	0.0693	0.115*	0.554 (6)
C4'	0.5616 (9)	−0.249 (3)	0.0778 (10)	0.111 (5)	0.554 (6)
H4'1	0.5798	−0.4026	0.0616	0.133*	0.554 (6)
H4'2	0.4954	−0.2037	0.0407	0.133*	0.554 (6)
C5'	0.5447 (6)	−0.2350 (14)	0.1783 (6)	0.082 (2)	0.554 (6)
H5'1	0.5157	−0.0869	0.1912	0.098*	0.554 (6)
H5'2	0.4909	−0.3471	0.1922	0.098*	0.554 (6)
C6'	0.6494 (6)	−0.2736 (13)	0.2392 (4)	0.0629 (18)	0.554 (6)
H6'1	0.6349	−0.2592	0.3027	0.075*	0.554 (6)
H6'2	0.6759	−0.4258	0.2299	0.075*	0.554 (6)

### Atomic displacement parameters (Å^2^)

**Table:**

	*U*^11^	*U*^22^	*U*^33^	*U*^12^	*U*^13^	*U*^23^
S1	0.0420 (4)	0.0551 (5)	0.0476 (4)	0.0008 (3)	0.0057 (3)	−0.0027 (3)
S2	0.0586 (5)	0.0585 (6)	0.0565 (5)	−0.0121 (3)	0.0090 (3)	0.0002 (3)
Na1	0.0465 (6)	0.0453 (8)	0.0609 (7)	0.0002 (4)	0.0053 (5)	−0.0005 (5)
O1	0.0534 (11)	0.0470 (13)	0.0608 (12)	−0.0039 (9)	−0.0015 (9)	0.0059 (10)
O2	0.0502 (10)	0.0497 (13)	0.0597 (12)	0.0060 (9)	0.0033 (8)	−0.0031 (10)
C1	0.0574 (15)	0.056 (2)	0.0512 (15)	−0.0111 (13)	0.0027 (12)	−0.0035 (14)
N1	0.062 (4)	0.052 (4)	0.047 (3)	−0.008 (3)	−0.001 (3)	0.000 (2)
C2	0.108 (6)	0.080 (7)	0.051 (4)	−0.026 (5)	−0.008 (4)	0.007 (4)
C3	0.104 (14)	0.107 (13)	0.079 (10)	−0.051 (11)	0.044 (10)	−0.030 (8)
C4	0.077 (8)	0.090 (9)	0.070 (6)	−0.033 (7)	0.003 (6)	−0.028 (7)
C5	0.070 (5)	0.070 (6)	0.088 (6)	−0.017 (5)	0.014 (4)	0.000 (5)
C6	0.053 (4)	0.086 (7)	0.061 (4)	−0.022 (5)	0.010 (4)	−0.010 (5)
N1'	0.052 (3)	0.056 (3)	0.047 (2)	−0.011 (2)	0.0082 (18)	−0.007 (2)
C2'	0.071 (4)	0.084 (5)	0.050 (3)	−0.020 (3)	0.016 (3)	−0.018 (3)
C3'	0.105 (12)	0.130 (13)	0.049 (6)	−0.041 (9)	−0.004 (7)	0.016 (7)
C4'	0.082 (8)	0.148 (14)	0.096 (7)	−0.042 (7)	−0.030 (6)	0.037 (7)
C5'	0.061 (4)	0.077 (6)	0.107 (6)	−0.020 (4)	0.007 (4)	−0.002 (4)
C6'	0.066 (4)	0.060 (5)	0.061 (4)	−0.027 (3)	0.000 (3)	−0.009 (3)

### Geometric parameters (Å, °)

**Table:**

S1—C1	1.726 (3)	C3—H3A	0.97
S1—Na1^i^	2.9644 (15)	C3—H3B	0.97
S1—Na1	3.0649 (15)	C4—C5	1.530 (18)
S2—C1	1.710 (3)	C4—H4A	0.97
Na1—O2	2.360 (3)	C4—H4B	0.97
Na1—O1	2.385 (2)	C5—C6	1.521 (14)
Na1—O1^ii^	2.416 (3)	C5—H5A	0.97
Na1—O2^iii^	2.515 (2)	C5—H5B	0.97
Na1—S1^iv^	2.9644 (15)	C6—H6A	0.97
Na1—Na1^ii^	3.490 (3)	C6—H6B	0.97
Na1—Na1^iii^	3.644 (3)	N1'—C2'	1.452 (6)
Na1—H12O	2.67 (3)	N1'—C6'	1.473 (7)
O1—Na1^ii^	2.416 (3)	C2'—C3'	1.495 (19)
O1—H11O	0.839 (17)	C2'—H2'1	0.97
O1—H12O	0.852 (17)	C2'—H2'2	0.97
O2—Na1^iii^	2.515 (2)	C3'—C4'	1.50 (3)
O2—H21O	0.834 (17)	C3'—H3'1	0.97
O2—H22O	0.856 (17)	C3'—H3'2	0.97
C1—N1'	1.368 (5)	C4'—C5'	1.512 (17)
C1—N1	1.403 (6)	C4'—H4'1	0.97
N1—C2	1.465 (9)	C4'—H4'2	0.97
N1—C6	1.489 (10)	C5'—C6'	1.507 (11)
C2—C3	1.47 (3)	C5'—H5'1	0.97
C2—H2A	0.97	C5'—H5'2	0.97
C2—H2B	0.97	C6'—H6'1	0.97
C3—C4	1.55 (3)	C6'—H6'2	0.97
			
C1—S1—Na1^i^	112.20 (10)	C3—C2—H2A	108.9
C1—S1—Na1	131.13 (10)	N1—C2—H2B	108.9
Na1^i^—S1—Na1	116.43 (4)	C3—C2—H2B	108.9
O2—Na1—O1	93.59 (8)	H2A—C2—H2B	107.8
O2—Na1—O1^ii^	169.15 (8)	C2—C3—C4	108.3 (14)
O1—Na1—O1^ii^	86.75 (8)	C2—C3—H3A	110
O2—Na1—O2^iii^	83.29 (8)	C4—C3—H3A	110
O1—Na1—O2^iii^	82.36 (7)	C2—C3—H3B	110
O1^ii^—Na1—O2^iii^	86.01 (8)	C4—C3—H3B	110
O2—Na1—S1^iv^	87.16 (7)	H3A—C3—H3B	108.4
O1—Na1—S1^iv^	166.91 (6)	C5—C4—C3	113.1 (13)
O1^ii^—Na1—S1^iv^	90.09 (7)	C5—C4—H4A	109
O2^iii^—Na1—S1^iv^	84.76 (6)	C3—C4—H4A	109
O2—Na1—S1	108.51 (7)	C5—C4—H4B	109
O1—Na1—S1	100.34 (7)	C3—C4—H4B	109
O1^ii^—Na1—S1	82.06 (7)	H4A—C4—H4B	107.8
O2^iii^—Na1—S1	167.57 (6)	C6—C5—C4	108.2 (9)
S1^iv^—Na1—S1	91.78 (4)	C6—C5—H5A	110.1
O2—Na1—Na1^ii^	136.28 (8)	C4—C5—H5A	110.1
O1—Na1—Na1^ii^	43.73 (6)	C6—C5—H5B	110.1
O1^ii^—Na1—Na1^ii^	43.02 (5)	C4—C5—H5B	110.1
O2^iii^—Na1—Na1^ii^	82.00 (7)	H5A—C5—H5B	108.4
S1^iv^—Na1—Na1^ii^	131.81 (6)	N1—C6—C5	110.1 (6)
S1—Na1—Na1^ii^	91.55 (6)	N1—C6—H6A	109.6
O2—Na1—Na1^iii^	43.27 (6)	C5—C6—H6A	109.6
O1—Na1—Na1^iii^	87.06 (7)	N1—C6—H6B	109.6
O1^ii^—Na1—Na1^iii^	126.00 (8)	C5—C6—H6B	109.6
O2^iii^—Na1—Na1^iii^	40.02 (5)	H6A—C6—H6B	108.2
S1^iv^—Na1—Na1^iii^	84.54 (5)	C1—N1'—C2'	124.6 (4)
S1—Na1—Na1^iii^	151.58 (5)	C1—N1'—C6'	122.6 (4)
Na1^ii^—Na1—Na1^iii^	111.83 (7)	C2'—N1'—C6'	111.9 (5)
O2—Na1—H12O	80.3 (6)	N1'—C2'—C3'	109.4 (7)
O1—Na1—H12O	18.4 (4)	N1'—C2'—H2'1	109.8
O1^ii^—Na1—H12O	97.4 (7)	C3'—C2'—H2'1	109.8
O2^iii^—Na1—H12O	68.4 (5)	N1'—C2'—H2'2	109.8
S1^iv^—Na1—H12O	151.4 (5)	C3'—C2'—H2'2	109.8
S1—Na1—H12O	116.5 (5)	H2'1—C2'—H2'2	108.2
Na1^ii^—Na1—H12O	56.0 (6)	C2'—C3'—C4'	116.5 (14)
Na1^iii^—Na1—H12O	68.7 (4)	C2'—C3'—H3'1	108.2
Na1—O1—Na1^ii^	93.25 (8)	C4'—C3'—H3'1	108.2
Na1—O1—H11O	129 (2)	C2'—C3'—H3'2	108.2
Na1^ii^—O1—H11O	97 (3)	C4'—C3'—H3'2	108.2
Na1—O1—H12O	100 (2)	H3'1—C3'—H3'2	107.3
Na1^ii^—O1—H12O	124 (2)	C3'—C4'—C5'	106.7 (11)
H11O—O1—H12O	114 (3)	C3'—C4'—H4'1	110.4
Na1—O2—Na1^iii^	96.71 (8)	C5'—C4'—H4'1	110.4
Na1—O2—H21O	121 (3)	C3'—C4'—H4'2	110.4
Na1^iii^—O2—H21O	96 (2)	C5'—C4'—H4'2	110.4
Na1—O2—H22O	121 (2)	H4'1—C4'—H4'2	108.6
Na1^iii^—O2—H22O	106 (2)	C6'—C5'—C4'	112.5 (7)
H21O—O2—H22O	111 (2)	C6'—C5'—H5'1	109.1
N1'—C1—N1	40.3 (3)	C4'—C5'—H5'1	109.1
N1'—C1—S2	117.2 (3)	C6'—C5'—H5'2	109.1
N1—C1—S2	117.4 (3)	C4'—C5'—H5'2	109.1
N1'—C1—S1	118.6 (3)	H5'1—C5'—H5'2	107.8
N1—C1—S1	116.2 (3)	N1'—C6'—C5'	110.6 (5)
S2—C1—S1	121.25 (16)	N1'—C6'—H6'1	109.5
C1—N1—C2	123.5 (5)	C5'—C6'—H6'1	109.5
C1—N1—C6	123.9 (6)	N1'—C6'—H6'2	109.5
C2—N1—C6	111.0 (6)	C5'—C6'—H6'2	109.5
N1—C2—C3	113.2 (13)	H6'1—C6'—H6'2	108.1
N1—C2—H2A	108.9		
			
C1—S1—Na1—O2	38.84 (16)	Na1^i^—S1—C1—S2	−10.8 (2)
Na1^i^—S1—Na1—O2	−135.11 (7)	Na1—S1—C1—S2	175.08 (10)
C1—S1—Na1—O1	−58.52 (16)	N1'—C1—N1—C2	−89.0 (9)
Na1^i^—S1—Na1—O1	127.53 (7)	S2—C1—N1—C2	11.7 (9)
C1—S1—Na1—O1^ii^	−143.72 (15)	S1—C1—N1—C2	166.8 (6)
Na1^i^—S1—Na1—O1^ii^	42.34 (6)	N1'—C1—N1—C6	75.0 (8)
C1—S1—Na1—O2^iii^	−160.0 (3)	S2—C1—N1—C6	175.6 (6)
Na1^i^—S1—Na1—O2^iii^	26.0 (3)	S1—C1—N1—C6	−29.3 (9)
C1—S1—Na1—S1^iv^	126.44 (14)	C1—N1—C2—C3	104.9 (12)
Na1^i^—S1—Na1—S1^iv^	−47.50 (6)	C6—N1—C2—C3	−60.8 (14)
C1—S1—Na1—Na1^ii^	−101.64 (15)	N1—C2—C3—C4	55 (2)
Na1^i^—S1—Na1—Na1^ii^	84.41 (5)	C2—C3—C4—C5	−53 (2)
C1—S1—Na1—Na1^iii^	44.63 (19)	C3—C4—C5—C6	54.8 (15)
Na1^i^—S1—Na1—Na1^iii^	−129.32 (11)	C1—N1—C6—C5	−105.1 (9)
O2—Na1—O1—Na1^ii^	169.13 (8)	C2—N1—C6—C5	60.6 (11)
O1^ii^—Na1—O1—Na1^ii^	0	C4—C5—C6—N1	−57.2 (11)
O2^iii^—Na1—O1—Na1^ii^	86.40 (8)	N1—C1—N1'—C2'	90.7 (8)
S1^iv^—Na1—O1—Na1^ii^	76.2 (3)	S2—C1—N1'—C2'	−10.6 (7)
S1—Na1—O1—Na1^ii^	−81.31 (7)	S1—C1—N1'—C2'	−171.6 (4)
Na1^iii^—Na1—O1—Na1^ii^	126.33 (8)	N1—C1—N1'—C6'	−77.5 (6)
O1—Na1—O2—Na1^iii^	−81.86 (8)	S2—C1—N1'—C6'	−178.7 (4)
O1^ii^—Na1—O2—Na1^iii^	9.6 (5)	S1—C1—N1'—C6'	20.2 (7)
O2^iii^—Na1—O2—Na1^iii^	0	C1—N1'—C2'—C3'	−113.5 (12)
S1^iv^—Na1—O2—Na1^iii^	85.05 (6)	C6'—N1'—C2'—C3'	55.7 (13)
S1—Na1—O2—Na1^iii^	175.98 (6)	N1'—C2'—C3'—C4'	−55 (2)
Na1^ii^—Na1—O2—Na1^iii^	−70.99 (11)	C2'—C3'—C4'—C5'	52.4 (19)
Na1^i^—S1—C1—N1'	149.5 (3)	C3'—C4'—C5'—C6'	−52.4 (15)
Na1—S1—C1—N1'	−24.6 (4)	C1—N1'—C6'—C5'	110.7 (7)
Na1^i^—S1—C1—N1	−164.9 (4)	C2'—N1'—C6'—C5'	−58.7 (9)
Na1—S1—C1—N1	21.0 (5)	C4'—C5'—C6'—N1'	57.7 (11)

Symmetry codes: (i) −*x*+3/2, *y*+1/2, −*z*+1; (ii) −*x*+1, −*y*, −*z*+1; (iii) −*x*+1, −*y*−1, −*z*+1; (iv) −*x*+3/2, *y*−1/2, −*z*+1.

### Hydrogen-bond geometry (Å, °)

**Table:**

*D*—H···*A*	*D*—H	H···*A*	*D*···*A*	*D*—H···*A*
O1—H11O···S2^v^	0.84 (2)	2.39 (2)	3.214 (2)	167 (3)
O1—H12O···S1^vi^	0.85 (2)	2.49 (2)	3.322 (3)	167 (3)
O2—H21O···S2^vi^	0.83 (2)	2.48 (2)	3.283 (2)	163 (3)
O2—H22O···S1^vii^	0.86 (2)	2.46 (2)	3.313 (2)	174 (3)

Symmetry codes: (v) *x*−1/2, −*y*+1/2, *z*; (vi) *x*−1/2, −*y*−1/2, *z*; (vii) *x*, *y*−1, *z*.
